# Gramene: Development and Integration of Trait and Gene Ontologies for Rice

**DOI:** 10.1002/cfg.156

**Published:** 2002-04

**Authors:** Pankaj Jaiswal, Doreen Ware, Junjian Ni, Kuan Chang, Wei Zhao, Steven Schmidt, Xiaokang Pan, Kenneth Clark, Leonid Teytelman, Samuel Cartinhour, Lincoln Stein, Susan McCouch

**Affiliations:** 1 Department of Plant Breeding Cornell University Ithaca NY 14853-1901 USA; 2 USDA-ARS Center for Agricultural Bioinformatics 626 Rhodes Hall Cornell Theory Center Ithaca NY 14853 USA; 3 Cold Spring Harbor Laboratory 1 Bungtown Road Cold Spring Harbor NY 11724 USA; 4 Department of Plant Breeding 418-Bradfield Hall Cornell University Ithaca NY 14853-1901 USA

## Abstract

Gramene (http://www.gramene.org/) is a comparative genome database for cereal crops and a community resource for rice. We are populating and curating Gramene with annotated rice (*Oryza sativa*) genomic sequence data and associated biological information including molecular markers, mutants, phenotypes, polymorphisms and Quantitative Trait Loci (QTL). In order to support queries across various data sets as well as across external databases, Gramene will employ three related controlled vocabularies. The specific goal of Gramene is, first to provide a Trait Ontology (TO) that can be used across the cereal crops to facilitate phenotypic comparisons both within and between the genera. Second, a vocabulary for plant anatomy terms, the Plant Ontology (PO) will facilitate the curation of morphological and anatomical feature information with respect to expression, localization of genes and gene products and the affected plant parts in a phenotype. The TO and PO are both in the early stages of development in collaboration with the International Rice Research Institute, TAIR and MaizeDB as part of the Plant Ontology Consortium. Finally, as part of another consortium comprising macromolecular databases from other model organisms, the Gene Ontology Consortium, we are annotating the confirmed and predicted protein entries from rice using both electronic and manual curation.


**Comparative and Functional Genomics**
*Comp Funct Genom* 2002; **3**: 132–136.
Published online 14 March 2002 in Wiley InterScience (www.interscience.wiley.com). **DOI:** 10.1002/cfg.156

**Conference Review**

### Gramene: development and integration of trait and gene ontologies for rice

Pankaj Jaiswal[1], Doreen Ware[3], Junjian Ni[1], Kuan Chang[1], Wei Zhao[3], Steven Schmidt[3], Xiaokang Pan[3], Kenneth Clark[3], Leonid Teytelman[3], Samuel Cartinhour[2], Lincoln Stein[3] and Susan McCouch[1]*

[1] *Department of Plant Breeding, Cornell University, Ithaca NY 14853-1901, USA*
[2] *USDA-ARS Center for Agricultural Bioinformatics, 626 Rhodes Hall, Cornell Theory Center, Ithaca, NY 14853, USA*
[3] *Cold Spring Harbor Laboratory, 1 Bungtown Road, Cold Spring Harbor, NY 11724, USA*

**Correspondence to:*
*Department of Plant Breeding, 418-Bradfield Hall, Cornell University, Ithaca NY 14853-1901, USA.*
*E-mail: srm4@cornell.edu*

### Abstract

**Gramene (http://www.gramene.org/) is a comparative genome database for cereal crops and a community resource for rice. We are populating and curating Gramene with annotated rice (*Oryza sativa*) genomic sequence data and associated biological information including molecular markers, mutants, phenotypes, polymorphisms and Quantitative Trait Loci (QTL). In order to support queries across various data sets as well as across external databases, Gramene will employ three related controlled vocabularies. The specific goal of Gramene is, first to provide a Trait Ontology (TO) that can be used across the cereal crops to facilitate phenotypic comparisons both within and between the genera. Second, a vocabulary for plant anatomy terms, the Plant Ontology (PO) will facilitate the curation of morphological and anatomical feature information with respect to expression, localization of genes and gene products and the affected plant parts in a phenotype. The TO and PO are both in the early stages of development in collaboration with the International Rice Research Institute, TAIR and MaizeDB as part of the Plant Ontology Consortium. Finally, as part of another consortium comprising macromolecular databases from other model organisms, the Gene Ontology Consortium, we are annotating the confirmed and predicted protein entries from rice using both electronic and manual curation.** **Copyright © 2002 John Wiley & Sons, Ltd.**

Received: 13 February 2002
Accepted: 14 February 2002

**Keywords: trait ontology; controlled vocabulary; plant ontology; gene ontology; comparative genomics; *Oryza sativa*; genome annotation; functional annotation**

### Introduction

In recent years, the world of biology has experienced a renaissance in terms of data generation, processivity and representation, based on large-scale genome sequencing and functional genomics efforts for a number of organisms. The rise in the quantity and diversity of information in the public and private databases makes it increasingly important to describe and classify objects and the relationships between them in meaningful ways. This presents two challenges in terms of database structure. One is to efficiently organize and integrate information within a database, and the second is to structure information to maximize interoperability across databases. The latter is increasingly important given that every database has a unique mandate and way of presenting information but at the same time, each database aims to address a broader user community. From the users perspective, a database search is an effort to retrieve information relevant to a specific topic or question. If a search initiated in one database is seamlessly extended to another database to broaden or deepen the coverage of the search, the confidence and satisfaction of the user is enhanced.

In this direction, at the molecular sequence level, an effort has been made by the Gene Ontology Consortium to develop shared structural vocabularies adequate for the annotation of gene products across organisms [3]. The concept of the GO has been widely accepted and successfully applied

Copyright © 2002 John Wiley & Sons, Ltd.

towards the annotation of the first few fully sequenced genomes including yeast [5], mouse [1] and *Drosophila* [6]. The conserved synteny among grass genomes suggests that rice genomic information can function as a window to the structure and function of genomes from other cereal crops. Comparative studies have revealed conserved synteny among grass genomes [7,11,15] and promoted the idea of using rice genome sequence as a template for comparative genome mapping in the grasses. The Gramene database evolved to meet this challenge and to serve as a community resource for rice [16]. As a representative plant database, Gramene (**http://www.gramene.org/**) has initiated an effort to carefully integrate the GO components of molecular function, biological process and cellular component for annotating gene products from rice. The functional annotation is extended by developing the Trait Ontology (TO) for rice to provide a framework for annotating the trait/phenotypic descriptions of rice mutants, strains, phenotypes, polymorphisms and QTLs. Gramene curators are also participating in the collaborative development of a Plant Ontology (PO) that will provide a structured vocabulary for plant anatomy terms (including morphology) and a framework for curating information on tissue specific expression and localization of genes, gene products and the affected plant parts in a phenotype.

### Functional annotation of rice gene products, mutants and phenotypes

A wide array of genetic information is contained in the large reservoirs of crop plant germplasm that have been accumulated over many decades. This information has been enriched by the historical familiarity of the agricultural community with the performance characteristics, crossing histories and environmental adaptation of crop species. Agricultural researchers have carefully recorded information on mutants, strains, phenotypes, polymorphisms and QTLs and many such studies involve associations between phenotypes and molecular markers. Association studies also hold promise for assessing correlations between specific genetic variants (SSRs, SNPs) and trait differences on a population level. The most commonly used approach in association genetics identifies differences and similarities in allele frequencies using sequence or molecular marker information from individuals with a particular phenotype and unrelated control individuals [4]. Along similar lines, a 'candidate gene' approach is helpful in associating QTLs with specific genes based on their location or function in the same or a different organism. In this approach, a gene may be considered a candidate underlying a QTL, if it is known to be located in a chromosomal position that is coincident with a QTL, or to be involved in a particular biochemical pathway, or have a predicted phenotypic effect that is similar to the observed QTL effect. Once identified, these gene 'candidates' can be examined in detail to determine whether they have a functional association with a QTL of interest [12]. As many genes are generally included within the chromosomal boundaries of a QTL and more than one may be functionally relevant to the trait in question, it remains difficult to provide convincing evidence from a database search, that a candidate gene is in fact the locus that conditions the trait under study. However, biologists can narrow down the search space considerably using this kind of database querying capability and those working with genetically less studied organisms can leverage the genomic information from model organisms.

Rice has a large, publicly available germplasm collection in excess of 120 000 accessions [8] and an active research community, reflected by a reservoir of more than 3000 genetically mapped DNA markers, more than 150 morphological mutants [13] and a genome that is predicted to encode between 30 000–60 000 genes [10]. This is just a beginning given that thousands of new mutants and phenotypes are being generated from newly funded functional genomics projects involving high throughput mutagenesis techniques. In order to better characterize the sequenced genes and provide a powerful approach to understand their association with observed phenotypes/traits, Gramene has initiated work on the functional annotation of rice gene products using automated and manual curation [16]. The curation of gene products (both confirmed and predicted) involves assigning the GO components of molecular function, biological process and localization (to a cellular component). Inferences consider the types of evidence, such as a direct enzyme assay, expression pattern, genetic or physical interaction, immuno-localization, phenotype assay or just the sequence similarity. The information on gene products is available at **http://www.gramene.org/perl/protein_search**. At present, the database carries electronic annotation of the GO

Copyright © 2002 John Wiley & Sons, Ltd.

associations kindly provided by the EBI, UK, for the rice entries.

In the rice mutant and phenotype (QTL) database, features associated with mutants and phenotypes will carry an annotation for the modified sequences, linked molecular markers, gene (allele) name, expression patterns of the mutant gene, etc. and their map position on the genetic or the physical maps. One of the specific goals of Gramene is to provide a Trait Ontology (TO) that will enable users to query for candidate genes from a target region on a rice chromosome based on phenotypic comparisons across the grasses. Each curated entry from the protein, mutant and phenotype database will carry an evidence code (**http://www.gramene.org/plant_ontology/evidence_codes.html**) describing the basis for the assertion of either the TO or the GO components and will be supported by the citations from the literature database available at **http://www.gramene.org/perl/pub_search**. The first release of the rice mutant database is expected by May 2002.

The following section will focus on the development and integration of the Trait Ontology.

### Trait Ontology concept

The Trait Ontology for rice is initially based upon INGER's Standard Evaluation System for Rice [9] and was reorganized to present a structured vocabulary of terms representing a trait as a 'distinguishable feature or characteristic, or a quality of character or a phenotypic feature of a developing or developed individual'. A couple of examples are 'plant height', which has variables of height as 'tall, dwarf and intermediate' or 'leaf color', with variables of color as 'light green, green, dark green, pale, yellow, etc.' with respect to a reference. Over the years, traits have been defined in numerous ways. A 'genetic trait' can be linked to heritable genetic markers; an 'agronomic trait' is of importance to agronomy; a 'morphological trait' involves a visible modification in a plant part; a 'physiological trait' is one that represents a response to an environmental factor such as temperature, light or nutrients; a 'developmental trait' is related to a temporal or spatial change in the plant body over time; a 'biochemical trait' is related to a change observed in a protein and is often associated with an alteration in its biochemical function.

The trait categories listed above are understandable but not distinct and are often complementary. For example, a genetic trait such as 'male sterility' (TO : 0000437) has an instance of 'cytoplasmic male sterility' (CMS), TO : 0000232. The CMS trait is of agronomic importance in hybrid breeding and seed production programs. CMS is known to result from a dysfunction of the mitochondria leading to unavailability of viable pollens. The two main functions of mitochondria are to perform 'oxidative phosphorylation' (GO : 0006119) and the 'tricaboxylic acid cycle' (GO : 0006099). The 'oxidative phosphorylation' function involves several macromolecular complexes that have been defined as the cellular components by the GO. These complexes are, 'complex I (NADH to ubiquinone)' (GO : 0006120), 'complex II (succinate to ubiquinone)' (GO : 0006121), 'complex III (ubiquinone to cytochrome c)' (GO : 0006122), and 'complex IV (reduction of $O_2$)' (GO : 0006123). These macro complexes are made up of several proteins, most of which are imported into the mitochondria from the cytoplasm (protein-mitochondrial targeting GO : 0006626). Mitochondrial dysfunction occurs due to improper interaction of the proteins in a complex and normal function can be restored by introduction of a different allele, also called the 'fertility restorer' gene (*rf*), which changes the genotype. The sterile phenotype involves modification in the structure of anther walls, the tapetum layer (which provides nourishment to developing pollen) and pollen grains (microspores) during microsporogenesis. The modifications are known to result in either non-dehiscent anthers or malnourished, nonviable pollens leading to sterility. In this example, it was impossible to describe the CMS trait exclusively in any single category of genetic/morphological/biochemical/agronomic trait. The current TO resolves the problem by allowing 'one to many' relationships.

### Trait Ontology development and integration

In the TO, traits are classified under two categories, i.e. epigenetic and genetic traits. An 'epigenetic trait' represents a 'distinguishable feature or characteristic, or a quality of character, or a phenotypic feature of a developing or developed individual' which has arisen as a result of heritable changes at structural level that may regulate the expression of the genes rather than differences in the DNA sequence of the genes [2,14]. This category will

Copyright © 2002 John Wiley & Sons, Ltd. 

cover all traits observed for the same genotype that are heritable but occur in response to changes in geographical, environmental or physiological conditions that permanently change the way genes are regulated.

The trait/phenotype observed in mutants/genotypes generated by modification of sequence represent the broad category of 'genetic trait'. The 'plant genetic trait' has two instances of 'whole plant related' and 'plant organ related' traits. However, adopting the paradigm defined by the GO [3], trait terms can have more than one parent. In Figure 1, an example of multiple child parent relationships is 'plant height' (TO : 0000207). It is an instance of 'height related trait' (TO : 0000171), which is an instance of 'stem related trait' (TO : 0000361), 'stature/vigor related trait' (TO : 0000133) and 'stem morphology' (TO : 0000219), thus showing structured, yet multiple parent child relationships. Each term will have an internationally accepted definition and an associated comment field carrying the information on how the trait is evaluated in rice. Once again taking the example of 'plant height' (TO : 0000207) the rice specific definition is: 'measure of height from soil surface to panicle base in centimeters'. The attributes of 'plant height' combined with the environment in which a variety is grown, determine the way rice variety is evaluated. This information will be presented as comments for the TO : 0000207. Under 'plant height' the comments are, a rice plant is called 'semi-dwarf', if the height is less than 110 cm for a lowland variety, or up to 90 cm for an upland variety, 'intermediate' if between 110–130 cm for a lowland variety or between 90–125 cm for an upland variety, or 'tall' if more than 130 cm for lowland or more than 125 cm for an upland variety.

The present effort in Gramene is focused mainly on developing a TO specific for rice that will provide a useful entry point for people familiar with this species in an agricultural context. We also aim to gain experience and see to what an extent TO can capture useful phenotype descriptors for other grasses. At present, we have successfully curated about 800 mutants from the literature representing diverse agronomic and morphological traits in rice, using the existent TO terms. The Trait Ontology (for rice) is available at **http://www.gramene.org/plant_ontology/**. By developing the Trait Ontology for rice, we gained some useful experience while curating rice mutants and now look forward to curating the ones representing

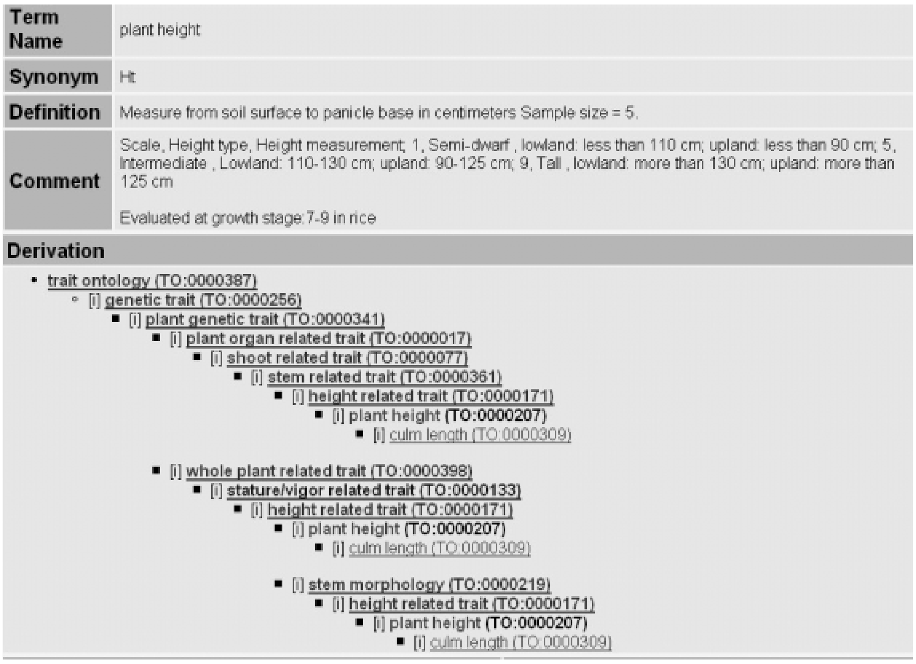

**Figure 1.** Modified view of the Ontology Browser at Gramene displaying the Summary for TO Term: plant height (TO : 0000207)

Copyright © 2002 John Wiley & Sons, Ltd.

complex traits. Our initial effort in development of TO is supported by the IRRI, ICIS, MaizeDB and CIMMYT.

The details about plant anatomy (including morphology) terms are given in the accompanying article (see The Plant Ontology Consortium, this issue) and the GO anatomy domains (**ftp://ftp.geneontology.org/pub/go/anatomy/**).

### Conclusion

The shared development and use of the controlled vocabularies such as GO, TO, and the PO, will help in structuring the datasets in Gramene on genes, gene products, mutants, strains, phenotypes, polymorphisms and quantitative trait loci. It will facilitate interoperability between databases by enabling us to link to the macromolecular databases from other model organisms such as yeast, fly, worm, mouse, rat, human and *Arabidopsis* at the gene product level. This will facilitate finding gene homologs and orthologs as possible candidate genes associated with phenotypes, polymorphisms and QTLs, and in clarifying their synteny relationships among grass genomes. In the future, it will permit a more complex annotation of genes and phenotypes and will provide researchers with an opportunity to perform sophisticated queries on the Gramene database by doing combination searches based on homeology and functional characteristics of genes. The development of plant specific vocabularies is open for community discussion and is encouraged by web based submission forms for suggestions, additions or modifications of various ontology terms. The form is available at **http://www.gramene.org/plant_ontology/submission/**.

### Acknowledgement

We kindly acknowledge Michael Ashburner (FlyBase, EBI, UK), Leonore Reiser (TAIR), the Gene Ontology Consortium, Richard Bruskiewich (IRRI), and Ed Coe, Mary Polacco and Leszek Vincent (MaizeDB) for their help on development of the Trait Ontology. We also acknowledge EBI for providing the electronic annotation of GO to rice associations. This work was supported by USDA CREES grant 00-52100-9622 and USDA ARS Specific Cooperative Agreement grant 58-1907-0-041.

### References

1. Blake JA, Richardson JE, Bult CJ, Kadin JA, Eppig JT. 2002. The Mouse Genome Database (MGD): the model organism database for the laboratory mouse. *Nucleic Acids Res* **30**: 113–115.
2. Chaudhury AM, Koltunow A, Payne T, *et al.* 2001. Control of early seed development. *Annu Rev Cell Dev Biol* **17**: 677–699.
3. GO Consortium. 2001. Creating the gene ontology resource: design and implementation. *Genome Res* **11**: 1425–1433.
4. Dekkers JC, Hospital F. 2002. The use of Molecular genetics in the improvement of agricultural populations. *Nat Rev Genet* **3**: 22–32.
5. Dwight SS, Harris MA, Dolinski K, *et al.* 2002. Saccharomyces Genome Database (SGD) provides secondary gene annotation using the Gene Ontology (GO). *Nucleic Acids Res* **30**: 69–72.
6. FlyBase. 2002. The FlyBase database of the *Drosophila* genome projects and community literature. *Nucleic Acids Res* **30**: 106–108.
7. Gale MD, Devos KM. 1998. Comparative genetics in the grasses. *Proc Natl Acad Sci U S A* **95**: 1971–1974.
8. ICIS. 2001. International Crop Information System. Ver 1.0. International Rice Research Institute: DAPO Box 7777, Metro Manila, Philippines; **http://www.cgiar.org/icis**
9. INGER. 1996. Standard Evaluation System for RICE. International Rice Research Institute: POB 933, Manila, Philippines; 1–52. **http://www.riceweb.org/ses/sesidx.htm**
10. Jun Y, Songnian H, Jun W, *et al.* 2001. A draft sequence of the rice (*Oryza sativa* ssp. *indica*). *Chinese Science Bulletin* **46**: 1937–1942
11. Keller B, Feuillet C. 2000. Colinearity and gene density in grass genomes. *Trends Plant Sci* **5**: 246–251.
12. Mauricio R. 2001. Mapping quantitative trait loci in plants: uses and caveats for evolutionary biology. *Nat Rev Genet* **2**: 370–381.
13. McCouch S. 1998. Toward a plant genomics initiative: thoughts on the value of cross- species and cross-genera comparisons in the grasses. *Proc Natl Acad Sci U S A* **95**: 1983–1985.
14. Pal C, Miklos I. 1999. Epigenetic inheritance, genetic assimilation and speciation. *J Theor Biol* **200**: 19–37.
15. Tarchini R, Biddle P, Wineland R, Tingey S, Rafalski A. 2000. The complete sequence of 340 kb of DNA around the rice Adh1-adh2 region reveals interrupted colinearity with maize chromosome 4. *Plant Cell* **12**: 381–391.
16. Ware D, Jaiswal P, Ni J, *et al.* 2002. Gramene: a resource for comparative grass genomics. *Nucleic Acids Res* **30**: 103–105.

Copyright © 2002 John Wiley & Sons, Ltd.
